# Crystal structure of 4-benzyl­carbamoyl-1-methyl­pyridin-1-ium iodide: an efficient multimodal anti­viral drug

**DOI:** 10.1107/S2056989017008155

**Published:** 2017-06-07

**Authors:** T. N. Drebushchak, Yu.A. Kryukov, A. I. Rogova, E. V. Boldyreva

**Affiliations:** a Institute of Solid State Chemistry and Mechanochemistry, SB RAS, ul. Kutateladze 18, Novosibirsk 630128, Russian Federation; b Novosibirsk State University, ul. Pirogova 2, Novosibirsk 630090, Russian Federation; c Institute for the Problems of the Technology of Energetic Materials, SB RAS, ul. Socialisticheskaia 1, Biisk 659322, Russian Federation

**Keywords:** crystal structure, drug, polymorphism

## Abstract

In the title compound, [*Me*C_5_H_4_NCONHCH_2_C_6_H_5_]I or C_14_H_15_N_2_O^+^·I^−^, a cation and an anion form an ionic pair linked by a strong N—H⋯I hydrogen bond. In the crystal, ionic pairs linked by weak C—H⋯I hydrogen bonds form infinite ribbons along the crystallographic *a* axis. Polymorphism screening varying crystallization solvents (water, acetone 90%–water, ethanol 90%–water, 2-propanol 90%–water, DMF, DMSO, methanol, aceto­nitrile) and conditions (solution temperature, heating and cooling protocols) did not reveal any other polymorphs than the one reported in this work.

## Abstract 2

Polymorph screening of the title compound, an efficient multimodal anti­viral drug, revealed only a single polymorph, for which the crystal structure is determined in this work.

## Chemical context   

**Figure 1:**
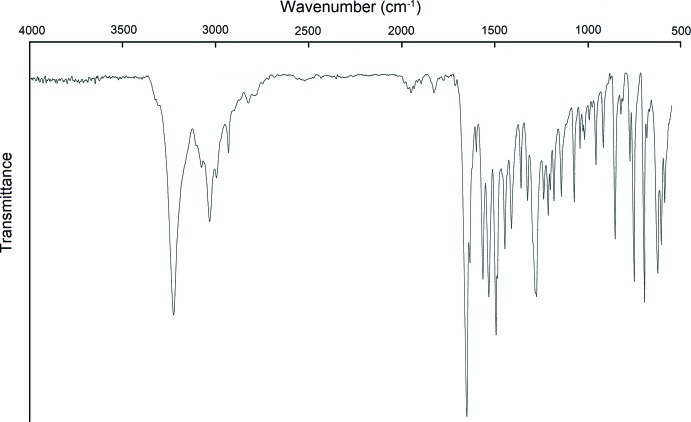
IR spectra of the title compound.

**Figure 2:**
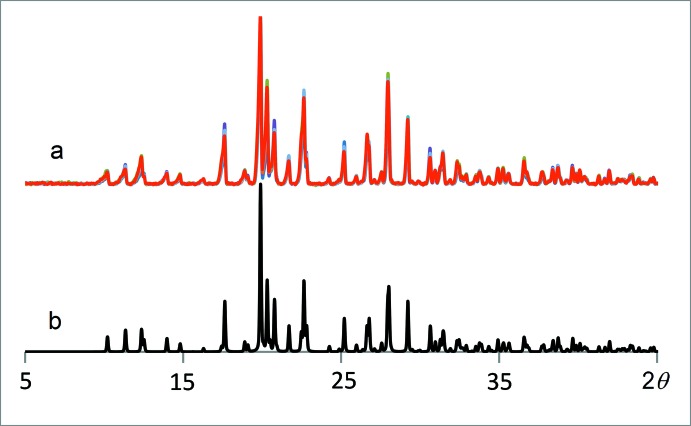
Powder diffraction patterns of the samples recrystallized from different solvents (an overlay) (*a*) and the diffraction pattern calculated for the structural model obtained based on single-crystal X-ray diffraction data in this work (*b*).

4-Benzyl­carbamoyl-1-methyl­pyridin-1-ium iodide, [*Me*C_5_H_4_NCONHCH_2_C_6_H_5_]I, is a multimodal anti­viral drug (Buhtiarova *et al.*, 2003 ▸; Frolov *et al.*, 2004 ▸). For pharmaceutical applications, it is of utmost importance to identify possible polymorphs (Bernstein, 2002 ▸; Brittain, 1999 ▸; Hilfiker, 2006 ▸), see also https://www.fda.gov/downloads/Drugs/Guidances/UCM072866.pdf; https://newdrugapprovals.org/2014/02/12/fda-guidance-on-polymorphic-compounds-in-generic-drugs/. Polymorphism screening varying crystallization solvents (water, acetone 90%–water, ethanol 90%–water, iso­propanol 90%–water, DMF, DMSO, MeOH, CH_3_CN) and conditions (solution temperature, heating and cooling protocols) did not reveal any other polymorphs than the one reported in this work as has been confirmed by DSC (METTLER TOLEDO DSC 822e, 5° min^−1^ in N_2_, samples 1/6–3/5 mg), IR spectroscopy (IR–FT spectrometer FT–801, spectroscopic resolution 0.5 cm^−1^ and systematic error ±0,05 cm^−1^; samples studied in KBr discs, 1.0 mg of substance in 200 mg of KBr; 4000–600 cm^−1^, and FTIR ATR spectrometer DigiLab Excalibur 3100, Varian spectrometer equipped with a MIRacle ATR accessory in the range 4000–600 cm^−1^ with resolution of 2 cm^−1^ without addition of KBr) and X-ray powder diffraction (STOE STADI MP diffractometer, CuKα_1_ radiation, curved Ge monochromator, transmission mode). The same thermal effect at the DSC curves related to sample melting at 464 K has been observed for all the samples. The position and relative intensities of the bands in the IR spectra were also the same (see section 5, Fig. 1 ▸). There were no differences between the IR spectra recorded with and without addition of KBr. The X-ray diffraction patterns were also the same for all the samples (Fig. 2 ▸) and matched the pattern calculated for the structural model based on single-crystal diffraction data (see next sections). *WinXPOW* (Stoe & Cie, 2011 ▸) was used to analyze the diffraction patterns.
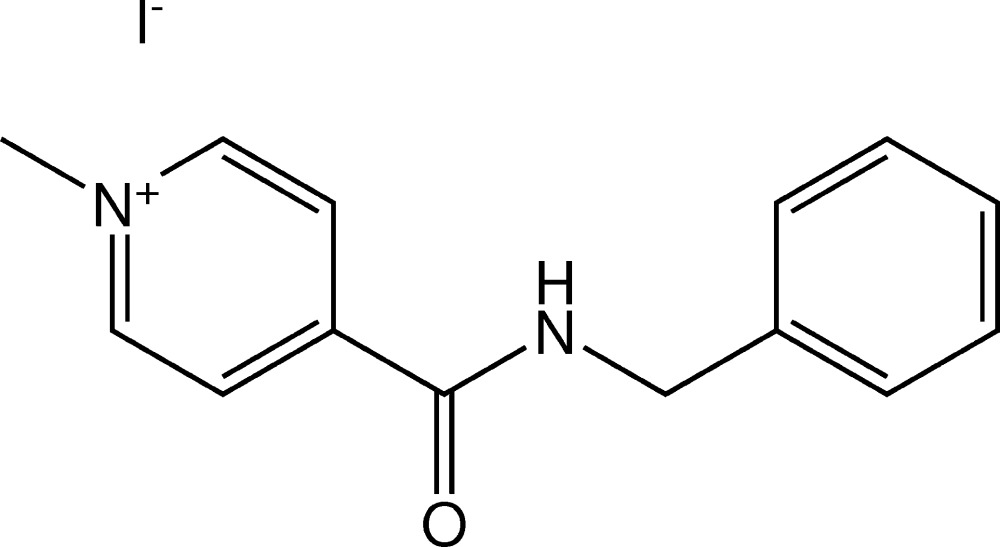


## Structural commentary   

**Figure 3:**
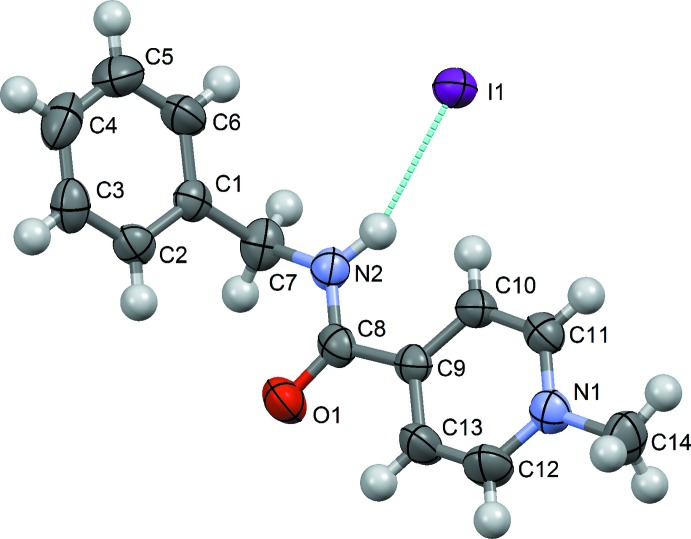
The mol­ecular structure of the title compound, showing the atom-labelling scheme. Displacement ellipsoids are drawn at the 50% probability level. H atoms are shown as spheres of arbitrary radius. The dotted line indicates the N—H⋯I^−^ hydrogen bond.

The asymmetric unit of the title compound contains a [*Me*C_5_H_4_NCONHCH_2_C_6_H_5_]^+^ cation and an I^−^ anion (Fig. 3 ▸). All the bond lengths and angles are within normal ranges. A cation and an anion form an ionic pair linked by a strong N2—H2⋯I1 hydrogen bond (Table 1 ▸). The central part of the mol­ecule (N2/C8/O1) and the pyridyl ring are located practically in the same plane [the average deviation of the atoms from the N1/N2/O1/C8–C13 plane is 0.015 (3) Å and the maximum deviation is 0.025 (3) Å]. The I^−^ anion is also close to this plane [at a distance of 0.504 (3) Å]. The dihedral angle between the pyridyl and benzene rings is 62.8 (1)°.

## Supra­molecular features   

**Figure 4:**
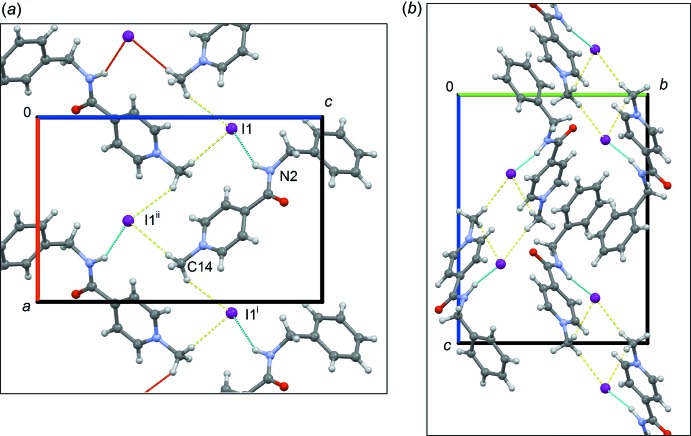
Crystal packing of the title compound, viewed (*a*) along the *b* axis and (*b*) along the *a* axis. The dotted lines indicate the hydrogen bonds, N—H⋯I (blue) and C—H⋯I (olive).

In addition to the strong NH⋯I hydrogen bond, two weak C—H⋯I hydrogen bonds are present in the crystal structure (Table 1 ▸, Fig. 4 ▸). Ionic pairs linked by these hydrogen bonds form infinite ribbons along the crystallographic *a* axis (Fig. 4 ▸). No hydrogen bonds link the ribbons with each other.

## Database survey   

**Figure 5:**
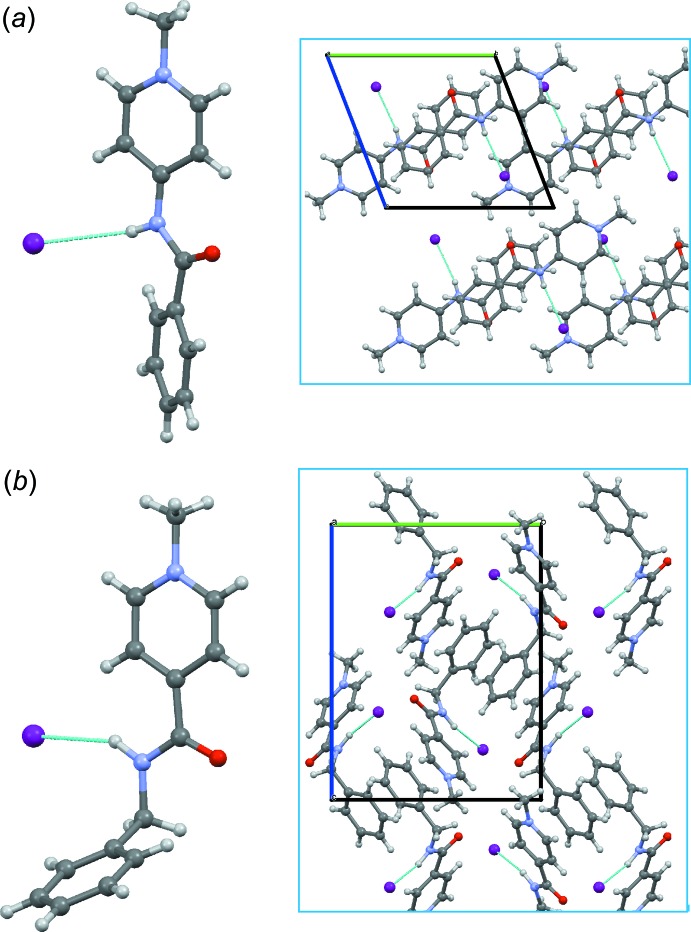
The mol­ecular structure and crystal packing of 4-benzoyl­amino-1-methyl­pyridinium iodide (*a*) and of the title compound (*b*).

No crystal structures containing the [*Me*C_5_H_4_NCONHCH_2_C_6_H_5_]^+^ cation could be found in the Cambridge Structural Database (CSD, Version 5.38, update November 2016; Groom *et al.*, 2016 ▸). A crystal structure of 4-benzoyl­amino-1-methyl­pyridinium iodide (CSD refcode ESESUS; Navarro *et al.*, 2016 ▸) is also formed by ionic pairs linked by a strong N—H⋯I hydrogen bond [the donor–acceptor distance is 3.675 (1) Å]. Similarly to the compound studied in this work, the organic cation of ESESUS also contains a benzene and an *N*-methyl­pyridine ring, with the N atom forming an N—H⋯I hydrogen bond in the centre of the cation (Fig. 5 ▸). Since the central part of the cation in the case of ESESUS is shorter than that of the title compound, the mol­ecular conformation is very different, as is the mol­ecular packing (Fig. 5 ▸).

## Synthesis and crystallization   

The title compound can be synthesized from *N*-benzyl­amide 4-pyridine­carb­oxy­lic acid C_5_H_4_NCONHCH_2_C_6_H_5_ and methyl iodide *Me*I in a 1:2 (Trinus *et al.*, 1994 ▸) or 1:1.2 (Buhtiarova *et al.*, 1997 ▸) molar ratio. *N*-Benzyl­amide 4-pyridine­carb­oxy­lic acid, in turn, was synthesized by the condensation of iso­nico­tinic acid C_5_H_4_NCOOH with benzyl­amine C_6_H_5_CH_2_NH_2_ taken in a 1:2 molar ratio (Trinus *et al.*, 1994 ▸).

12.31 g (0.1 mol) of isonicotinic acid were added with constant stirring over a period of one hour to 12.86 g (0.12 mol) of benzyl­amine heated to 413 K. After all of the isonicotinic acid had been added, the mixture was heated steadily to 493–503 K. After water and the excess of benzamine had been distilled, the residue was cooled to 373–383 K and added on stirring to 100 ml of toluene. The hot solution was filtered and cooled to 288 K. After cooling, the precipitate was filtered, washed on the filter with 20 ml of toluene and dried in the air at ambient temperature. The yield was 18.57 g (0.0875 mol; 87.5%) (Sysoljatin *et al.*, 2011 ▸).

18.57 g (0.0875 mol) of *N*-benzyl­amide 4-pyridine­carb­oxy­lic acid were added to 110 ml of acetone with stirring. After the dissolution was complete, 14.9 g (0.105 mol) of methyl iodide *Me*I were added and the reaction mixture kept at 323 K for five h after which it was cooled to 283–288 K and filtered. The precipitate was washed on the filter with 50 ml of acetone and dried in the air. The yield was 24.65 g (0.0696 mol; 79.5%) (Sysoljatin *et al.*, 2011 ▸).

Calculated for C_14_H_15_N_2_OI: C, 47.46; H, 4.21: N, 7.91; O, 4.52. Found: C, 47.31; H, 4.13: N, 7.62; O, 4.35. T_melt._ 464 K. IR spectrum (cm^−1^): 611.27, 631.44, 703.72, 777.41, 759.32, 860.4, 920.77, 960.85, 1020.8, 1078.2, 1147.6, 1187.8, 1218.8, 1285.4, 1329.8, 1416.4, 1452.5, 1505.1, 1541.1, 1571.6, 1663.7, 1641.4, 1828.1, 1950.9, 2828.6, 2936.6, 3040.4, 3237.6. ^1^H NMR (400 MHz, DMSO-*d*_6_, p.p.m.): δ = 4.40 (*s*, 3H, CH_3_), 4.55 (*d*, 2H, CH_2_), 7.22–7.45 (*m*, 5H, *Ar*), 8.44 (*d*, 2H, *Py*), 9.19 (*d*, 2H, *Py*), 9.78 (*s*, H, NH). ^13^C–^1^H NMR (100 MHz, DMSO-*d*_6_, p.p.m.): δ = 43.89 (CH_2_), 49.00 (CH_3_), 125.95, 147.06, 148.20 (*Py*), 127.65, 128.01, 128.92, 139.18 (*Ar*), 162.63 (C=O).

The pharmaceutical substance was obtained by recrystal­lization from an aqueous solution with activated carbon (Sysoljatin *et al.*, 2011 ▸). 5.0 g (0.014 mol) of *N*-methyl-4-benzyl­carbamido­pyridinium iodide were dissolved in 6 ml of water at 363 K and 0.15 g (3.0%) of activated carbon added. After the complete dissolution of the compound, the activated carbon was removed by filtering, and the solution was cooled to 283 K. After stirring for one hour, the precipitate formed was filtered through a paper filter (white band), washed with 10 ml of acetone and dried at 373 K. Yield 4.71 g (0.0133 mol; 94.3%).

## Refinement   

Crystal data, data collection and structure refinement details are summarized in Table 2 ▸. The amine hydrogen atom bound to N2 was located in the difference maps and refined isotropically. All other hydrogen atoms were positioned geom­etrically and refined with a riding model [C—H = 0.93–0.97 Å; *U*_iso_(H) = 1.2–1.5*U*_eq_(C)].

## Supplementary Material

Crystal structure: contains datablock(s) I. DOI: 10.1107/S2056989017008155/rk2435sup1.cif

Structure factors: contains datablock(s) I. DOI: 10.1107/S2056989017008155/rk2435Isup2.hkl

Supporting information file. DOI: 10.1107/S2056989017008155/rk2435Isup3.cml

CCDC reference: 1553547

Additional supporting information:  crystallographic information; 3D view; checkCIF report

## Figures and Tables

**Table 1:** Hydrogen-bond geometry (Å, °)

*D*—H⋯*A*	*D*—H	H⋯*A*	*D*⋯*A*	*D*—H⋯*A*
N2—H2⋯I1	0.98 (6)	2.68 (6)	3.563 (3)	150 (5)
C14—H14*A*⋯I1^i^	0.96	3.08	4.018 (5)	168
C14—H14*C*⋯I1^ii^	0.96	3.06	3.919 (5)	150

Symmetry codes: (i) 
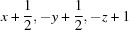
; (ii) 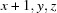.

**Table 2:** Experimental details

Crystal data	
Chemical formula	C_14_H_15_N_2_O^+^·I^−^
*M*_r_	354.18
Crystal system, space group	Orthorhombic, *P*2_1_2_1_2_1_
Temperature (K)	295
*a*, *b*, *c* (Å)	9.2867 (2), 10.8741 (2), 14.3038 (3)
*V* (Å^3^)	1444.46 (5)
*Z*	4
Radiation type	Mo *K*α
μ (mm^−1^)	2.21
Crystal size (mm)	0.25 × 0.17 × 0.07

Data collection	
Diffractometer	Rigaku OD Xcalibur, Ruby, Gemini ultra
Absorption correction	Multi-scan (*CrysAlis PRO*; Rigaku OD, 2015 ▸)
*T*_min_, *T*_max_	0.910, 1.000
No. of measured, independent and observed [*I* > 2σ(*I*)] reflections	18978, 3388, 3115
*R*_int_	0.033
(sin θ/λ)_max_ (Å^−1^)	0.666

Refinement	
*R*[*F* ^2^ > 2σ(*F* ^2^)], *wR*(*F* ^2^), *S*	0.024, 0.053, 1.08
No. of reflections	3388
No. of parameters	168
H-atom treatment	H atoms treated by a mixture of independent and constrained refinement
Δρ_max_, Δρ_min_ (e Å^−3^)	0.53, −0.35
Absolute structure	Flack *x* determined using 1207 quotients [(*I* ^+^)−(*I* ^−^)]/[(*I* ^+^)+(*I* ^−^)] (Parsons *et al.*, 2013 ▸)
Absolute structure parameter	−0.033 (11)

Computer programs: *CrysAlis PRO* (Rigaku OD, 2015 ▸), *SHELXS97* (Sheldrick, 2008 ▸), *SHELXL2014* (Sheldrick, 2015 ▸), *Mercury* (Macrae *et al.*, 2006 ▸), *WinGX* (Farrugia, 2012 ▸) and *publCIF* (Westrip, 2010 ▸).

## supplementary crystallographic information

### Crystal data

**Table:**

C_14_H_15_N_2_O^+^·I^−^	*D*_x_ = 1.629 Mg m^−^^3^
*M**_r_* = 354.18	Melting point: 464(2) K
Orthorhombic, *P*2_1_2_1_2_1_	Mo *K*α radiation, λ = 0.71073 Å
*a* = 9.2867 (2) Å	Cell parameters from 9574 reflections
*b* = 10.8741 (2) Å	θ = 2.4–26.3°
*c* = 14.3038 (3) Å	µ = 2.21 mm^−^^1^
*V* = 1444.46 (5) Å^3^	*T* = 295 K
*Z* = 4	Block, yellow
*F*(000) = 696	0.25 × 0.17 × 0.07 mm

### Data collection

**Table:**

Rigaku OD Xcalibur, Ruby, Gemini ultra diffractometer	3388 independent reflections
Radiation source: fine-focus sealed X-ray tube, Enhance (Mo) X-ray Source	3115 reflections with *I* > 2σ(*I*)
Graphite monochromator	*R*_int_ = 0.033
Detector resolution: 10.3457 pixels mm^-1^	θ_max_ = 28.3°, θ_min_ = 2.4°
ω scans	*h* = −12→12
Absorption correction: multi-scan (*CrysAlis PRO*; Rigaku OD, 2015)	*k* = −14→13
*T*_min_ = 0.910, *T*_max_ = 1.000	*l* = −18→18
18978 measured reflections	

### Refinement

**Table:**

Refinement on *F*^2^	Secondary atom site location: difference Fourier map
Least-squares matrix: full	Hydrogen site location: mixed
*R*[*F*^2^ > 2σ(*F*^2^)] = 0.024	H atoms treated by a mixture of independent and constrained refinement
*wR*(*F*^2^) = 0.053	*w* = 1/[σ^2^(*F*_o_^2^) + (0.0267*P*)^2^ + 0.0479*P*] where *P* = (*F*_o_^2^ + 2*F*_c_^2^)/3
*S* = 1.08	(Δ/σ)_max_ < 0.001
3388 reflections	Δρ_max_ = 0.53 e Å^−^^3^
168 parameters	Δρ_min_ = −0.35 e Å^−^^3^
0 restraints	Absolute structure: Flack *x* determined using 1207 quotients [(*I*^+^)-(*I*^-^)]/[(*I*^+^)+(*I*^-^)] (Parsons *et al.*, 2013)
Primary atom site location: structure-invariant direct methods	Absolute structure parameter: −0.033 (11)

### Special details

**Table:**

Geometry. All s.u.'s (except the s.u. in the dihedral angle between two l.s. planes) are estimated using the full covariance matrix. The cell s.u.'s are taken into account individually in the estimation of s.u.'s in distances, angles and torsion angles; correlations between s.u.'s in cell parameters are only used when they are defined by crystal symmetry. An approximate (isotropic) treatment of cell s.u.'s is used for estimating s.u.'s involving l.s. planes.

### Fractional atomic coordinates and isotropic or equivalent isotropic displacement parameters (Å^2^)

**Table:**

	*x*	*y*	*z*	*U*_iso_*/*U*_eq_	
C1	0.1886 (4)	0.0803 (3)	0.9728 (2)	0.0423 (8)	
C2	0.2939 (4)	0.0665 (4)	1.0407 (3)	0.0475 (9)	
H1	0.3631	0.0054	1.0338	0.057*	
C3	0.2982 (5)	0.1404 (4)	1.1172 (3)	0.0583 (11)	
H3	0.3702	0.1296	1.1617	0.070*	
C4	0.1975 (6)	0.2307 (5)	1.1293 (3)	0.0739 (13)	
H4	0.2012	0.2817	1.1814	0.089*	
C5	0.0909 (6)	0.2452 (5)	1.0639 (4)	0.0805 (16)	
H5	0.0210	0.3054	1.0723	0.097*	
C6	0.0869 (5)	0.1713 (4)	0.9858 (3)	0.0654 (12)	
H6	0.0149	0.1827	0.9414	0.078*	
C7	0.1838 (5)	−0.0028 (4)	0.8892 (3)	0.0555 (10)	
H7A	0.2008	−0.0868	0.9093	0.067*	
H7B	0.0882	0.0006	0.8619	0.067*	
C8	0.4168 (4)	−0.0275 (3)	0.8098 (3)	0.0456 (8)	
C9	0.5104 (4)	0.0078 (3)	0.7283 (3)	0.0407 (8)	
C10	0.4765 (4)	0.0948 (4)	0.6614 (3)	0.0477 (9)	
H10	0.3906	0.1385	0.6656	0.057*	
C11	0.5694 (5)	0.1168 (4)	0.5888 (3)	0.0504 (9)	
H11	0.5455	0.1751	0.5439	0.060*	
C12	0.7303 (5)	−0.0274 (5)	0.6477 (3)	0.0677 (13)	
H12	0.8177	−0.0686	0.6432	0.081*	
C13	0.6423 (5)	−0.0514 (5)	0.7198 (3)	0.0627 (12)	
H13	0.6700	−0.1084	0.7648	0.075*	
C14	0.7913 (5)	0.0782 (5)	0.5020 (3)	0.0705 (13)	
H14A	0.7477	0.1364	0.4602	0.106*	
H14B	0.8087	0.0025	0.4694	0.106*	
H14C	0.8810	0.1107	0.5246	0.106*	
N1	0.6942 (4)	0.0555 (3)	0.5815 (2)	0.0494 (8)	
N2	0.2902 (3)	0.0295 (3)	0.8172 (2)	0.0475 (7)	
H2	0.262 (7)	0.085 (6)	0.766 (4)	0.12 (2)*	
O1	0.4598 (4)	−0.1053 (3)	0.86557 (19)	0.0634 (8)	
I1	0.06127 (3)	0.22276 (3)	0.68164 (2)	0.05375 (9)	

### Atomic displacement parameters (Å^2^)

**Table:**

	*U*^11^	*U*^22^	*U*^33^	*U*^12^	*U*^13^	*U*^23^
C1	0.0396 (19)	0.0438 (18)	0.0435 (18)	−0.0051 (15)	0.0082 (17)	0.0088 (16)
C2	0.0350 (19)	0.057 (2)	0.050 (2)	0.0010 (16)	0.0021 (18)	0.0085 (19)
C3	0.055 (3)	0.075 (3)	0.044 (2)	−0.014 (2)	0.002 (2)	0.003 (2)
C4	0.103 (4)	0.069 (3)	0.050 (2)	−0.011 (3)	0.018 (3)	−0.011 (2)
C5	0.096 (4)	0.069 (3)	0.076 (3)	0.033 (3)	0.016 (3)	0.000 (2)
C6	0.059 (3)	0.078 (3)	0.059 (3)	0.023 (2)	−0.002 (2)	0.015 (2)
C7	0.052 (2)	0.063 (3)	0.051 (2)	−0.015 (2)	0.002 (2)	−0.0028 (19)
C8	0.051 (2)	0.0456 (19)	0.0401 (18)	−0.0024 (16)	−0.011 (2)	−0.0033 (16)
C9	0.0420 (19)	0.042 (2)	0.0382 (19)	−0.0002 (15)	−0.0065 (16)	−0.0043 (15)
C10	0.048 (2)	0.047 (2)	0.048 (2)	0.0084 (16)	−0.0015 (17)	−0.0013 (16)
C11	0.054 (2)	0.048 (2)	0.049 (2)	0.002 (2)	−0.004 (2)	0.0057 (16)
C12	0.046 (3)	0.087 (4)	0.070 (3)	0.020 (2)	0.002 (2)	0.012 (3)
C13	0.056 (3)	0.076 (3)	0.055 (2)	0.021 (2)	−0.002 (2)	0.018 (2)
C14	0.058 (3)	0.097 (4)	0.057 (3)	−0.001 (3)	0.010 (2)	0.004 (3)
N1	0.0408 (17)	0.061 (2)	0.0463 (17)	−0.0012 (15)	−0.0045 (15)	−0.0002 (15)
N2	0.0472 (18)	0.0553 (19)	0.0400 (16)	0.0010 (14)	0.0006 (18)	−0.0012 (17)
O1	0.068 (2)	0.0664 (18)	0.0562 (15)	0.0077 (17)	−0.0044 (16)	0.0181 (15)
I1	0.05146 (14)	0.05593 (15)	0.05386 (14)	0.01142 (12)	−0.00363 (13)	−0.00252 (12)

### Geometric parameters (Å, º)

**Table:**

C1—C6	1.381 (6)	C8—C9	1.504 (5)
C1—C2	1.387 (5)	C9—C10	1.383 (5)
C1—C7	1.500 (5)	C9—C13	1.389 (6)
C2—C3	1.358 (6)	C10—C11	1.371 (5)
C2—H1	0.9300	C10—H10	0.9300
C3—C4	1.367 (7)	C11—N1	1.341 (5)
C3—H3	0.9300	C11—H11	0.9300
C4—C5	1.371 (7)	C12—C13	1.342 (6)
C4—H4	0.9300	C12—N1	1.349 (5)
C5—C6	1.376 (7)	C12—H12	0.9300
C5—H5	0.9300	C13—H13	0.9300
C6—H6	0.9300	C14—N1	1.472 (5)
C7—N2	1.469 (5)	C14—H14A	0.9600
C7—H7A	0.9700	C14—H14B	0.9600
C7—H7B	0.9700	C14—H14C	0.9600
C8—O1	1.229 (4)	N2—H2	0.98 (6)
C8—N2	1.334 (5)		
			
C6—C1—C2	117.7 (4)	C10—C9—C13	117.2 (4)
C6—C1—C7	121.3 (4)	C10—C9—C8	125.5 (3)
C2—C1—C7	121.0 (4)	C13—C9—C8	117.3 (4)
C3—C2—C1	121.4 (4)	C11—C10—C9	120.0 (4)
C3—C2—H1	119.3	C11—C10—H10	120.0
C1—C2—H1	119.3	C9—C10—H10	120.0
C2—C3—C4	120.4 (4)	N1—C11—C10	121.1 (4)
C2—C3—H3	119.8	N1—C11—H11	119.4
C4—C3—H3	119.8	C10—C11—H11	119.4
C3—C4—C5	119.4 (4)	C13—C12—N1	121.2 (4)
C3—C4—H4	120.3	C13—C12—H12	119.4
C5—C4—H4	120.3	N1—C12—H12	119.4
C4—C5—C6	120.4 (4)	C12—C13—C9	120.9 (4)
C4—C5—H5	119.8	C12—C13—H13	119.5
C6—C5—H5	119.8	C9—C13—H13	119.5
C5—C6—C1	120.6 (4)	N1—C14—H14A	109.5
C5—C6—H6	119.7	N1—C14—H14B	109.5
C1—C6—H6	119.7	H14A—C14—H14B	109.5
N2—C7—C1	113.2 (3)	N1—C14—H14C	109.5
N2—C7—H7A	108.9	H14A—C14—H14C	109.5
C1—C7—H7A	108.9	H14B—C14—H14C	109.5
N2—C7—H7B	108.9	C11—N1—C12	119.4 (4)
C1—C7—H7B	108.9	C11—N1—C14	120.4 (3)
H7A—C7—H7B	107.8	C12—N1—C14	120.2 (4)
O1—C8—N2	123.7 (4)	C8—N2—C7	122.5 (4)
O1—C8—C9	119.4 (4)	C8—N2—H2	118 (4)
N2—C8—C9	116.9 (3)	C7—N2—H2	119 (4)
			
C6—C1—C2—C3	−0.6 (6)	C13—C9—C10—C11	2.1 (6)
C7—C1—C2—C3	−179.3 (4)	C8—C9—C10—C11	−178.4 (3)
C1—C2—C3—C4	0.3 (6)	C9—C10—C11—N1	−0.4 (6)
C2—C3—C4—C5	0.6 (7)	N1—C12—C13—C9	0.5 (8)
C3—C4—C5—C6	−1.2 (8)	C10—C9—C13—C12	−2.2 (7)
C4—C5—C6—C1	0.9 (8)	C8—C9—C13—C12	178.2 (4)
C2—C1—C6—C5	0.0 (6)	C10—C11—N1—C12	−1.4 (6)
C7—C1—C6—C5	178.7 (4)	C10—C11—N1—C14	178.7 (4)
C6—C1—C7—N2	103.1 (4)	C13—C12—N1—C11	1.3 (7)
C2—C1—C7—N2	−78.2 (5)	C13—C12—N1—C14	−178.7 (5)
O1—C8—C9—C10	−179.0 (4)	O1—C8—N2—C7	−5.2 (6)
N2—C8—C9—C10	0.6 (5)	C9—C8—N2—C7	175.1 (3)
O1—C8—C9—C13	0.5 (5)	C1—C7—N2—C8	97.8 (4)
N2—C8—C9—C13	−179.8 (4)		

### Hydrogen-bond geometry (Å, º)

**Table:**

*D*—H···*A*	*D*—H	H···*A*	*D*···*A*	*D*—H···*A*
N2—H2···I1	0.98 (6)	2.68 (6)	3.563 (3)	150 (5)
C14—H14*A*···I1^i^	0.96	3.08	4.018 (5)	168
C14—H14*C*···I1^ii^	0.96	3.06	3.919 (5)	150

Symmetry codes: (i) *x*+1/2, −*y*+1/2, −*z*+1; (ii) *x*+1, *y*, *z*.
